# E-Coaching for parents of young children with autism spectrum disorder (ASD): study protocol, feasability and preliminary efficacy

**DOI:** 10.1192/j.eurpsy.2025.522

**Published:** 2025-08-26

**Authors:** C. Peter, M.-P. Antoniou, E. Antonietti, E. Bucaille, J. Almeida Osório, B. Rodríguez-Herreros, N. Chabane

**Affiliations:** 1 CHUV, Lausanne, Switzerland

## Abstract

**Introduction:**

Autism spectrum disorder (ASD) is a neurodevelopmental disorder that highly impacts children’s development, representing a significant challenge in pediatric healthcare. Parents of children with ASD are nowadays considered as real partners in their children’s care. Several parent-mediated interventions (PMIs) have proven to produce sustained improvements in autism symptomatology and social communication. However, widespread access to this type of intervention is still very limited mostly due to geographic and logistic constraints. The use of technology is therefore increasingly considered with the use of videoconferencing and online training modules. In this context, our team developed a novel parental coaching via E-learning (E-coaching) intended for parents of pre-school children with ASD.

**Objectives:**

The ongoing randomized controlled trial aims to evaluate the feasibility and preliminary efficacy of our E-Coaching program compared to a standard coaching and a control group with no PMI.

**Methods:**

The present study is a monocentric randomized controlled trial with three arms (E-coaching, Standard coaching, Control) of 33 children (N=99). Feasibility was assessed across recruitment, acceptability and implementation using semi-structured interviews. The primary outcome will be the quality of parent-child interaction, measured using a range of behavioral observations and by monitoring parent and child gaze using two head-mounted eye-tracking systems during semi-structured standardized play sessions. Secondary outcomes will include child’s developmental level through neuropsychological testing, and parental wellbeing through several standardized parent-report questionnaires.

**Results:**

We present preliminary evidence supporting the feasibility and acceptability of the intervention, with participants reporting positive benefits on parent-child interaction. Preliminary observational data on the first families provided support for an improvement on parent-child interaction immediately after the end of the E-coaching intervention, as well as an increase of parental wellbeing.

**Conclusions:**

We found initial feasibility for our E-coaching program, suggesting that parent-mediated E-learning interventions may be a promising format to implement with ASD families. Further evaluation to assess efficacy of the intervention is warranted and underway. If validated, E-coaching will enable us to reach a larger number of families and to have an early and meaningful impact on the developmental trajectory of these children, and on their quality of life.

**Disclosure of Interest:**

None Declared

